# Signal regression in frequency-domain diffuse optical tomography to remove superficial signal contamination

**DOI:** 10.1117/1.NPh.8.1.015013

**Published:** 2021-03-31

**Authors:** Joshua D. Veesa, Hamid Dehghani

**Affiliations:** University of Birmingham, School of Computer Science, Birmingham, United Kingdom

**Keywords:** frequency domain, high density diffuse optical tomography, signal regression, superficial signal contamination, functional near-infrared imaging

## Abstract

**Significance:** Signal contamination is a major hurdle in functional near-infrared spectroscopy (fNIRS) of the human head as the NIR signal is contaminated with the changes corresponding to superficial tissue, therefore occluding the functional information originating from the cerebral region. For continuous wave, this is generally handled through linear regression of the shortest source-detector (SD) distance intensity measurement from all of the signals. Although phase measurements utilizing frequency domain (FD) provide deeper tissue sampling, the use of the shortest SD distance phase measurement for regression of superficial signal contamination can lead to misleading results, therefore suppressing cortical signals.

**Aim:** An approach for FD fNIRS that utilizes a short-separation intensity signal directly to regress both intensity and phase measurements, providing a better regression of superficial signal contamination from both data-types, is proposed.

**Approach:** Simulated data from realistic models of the human head are used, and signal regression using both intensity and phase-based components of the FD fNIRS is evaluated.

**Results:** Intensity-based phase regression achieves a suppression of superficial signal contamination by 68% whereas phase-based phase regression is only by 13%. Phase-based phase regression is also shown to generate false-positive signals from the cortex, which are not desirable.

**Conclusions:** Intensity-based phase regression provides a better methodology for minimizing superficial signal contamination in FD fNIRS.

## 1 Introduction

Continuous wave (CW) functional near-infrared spectroscopy (fNIRS)-based optical imaging is a neuroimaging technique used to non-invasively monitor functional activity in the brain by tomographic reconstruction of the hemodynamic activity. These reconstructions can be used in clinical applications such as patient monitoring,^1^ psychology studies,^2^^,^^3^ and functional brain mapping.^4^ It is a relatively inexpensive technology and nonionizing in nature as compared with the alternative neuroimaging tools such as magnetic resonance imaging, computed tomography, or positron emission tomography. CW-NIRS-based imaging relies on the measurements of the light intensity, using multi-distance overlapping source-detector (SD) channels. NIR light sources placed on the scalp emit light into the head that traverses through the scalp, skull, cerebro-spinal fluid (CSF), and cerebral regions, and the back-scattered light is measured by detectors placed on the scalp at certain distances from the sources. The spectrally varying optical properties of the medium that contribute to variation of the measured light intensities are then reconstructed using a diffusion theory-based inverse model, from which 3D recovery of optical properties of the medium is often termed as diffuse optical tomography (DOT).

A major challenge to any fNIRS-based technique monitoring cerebral hemodynamic activity is the contamination of the NIR signal with hemodynamic changes arising in superficial regions, namely, skin and scalp.^1^^,^^5^ This has been shown to be reduced significantly for CW-NIRS by incorporating short SD distance (about 10 to 15 mm) in the measurement setup.^6^ As the short-distance measurements mainly contain the superficial signal, it is directly regressed from all of the remaining measurements to reduce the superficial tissue contamination from the data. In DOT, the overlapping measurements of the multi-SD setup, together with the varying depth sensitivity based on SD distances, allow the spatial reconstruction of the hemodynamic changes, therefore, potentially separating the contributions from deep and shallow regions.^7^ However, it is common practice to apply superficial signal regression with the DOT algorithm prior to the reconstruction step to significantly improve the 3D spatial recovery and the recovered contrast of focal activations.^8^

**Fig. 1:**
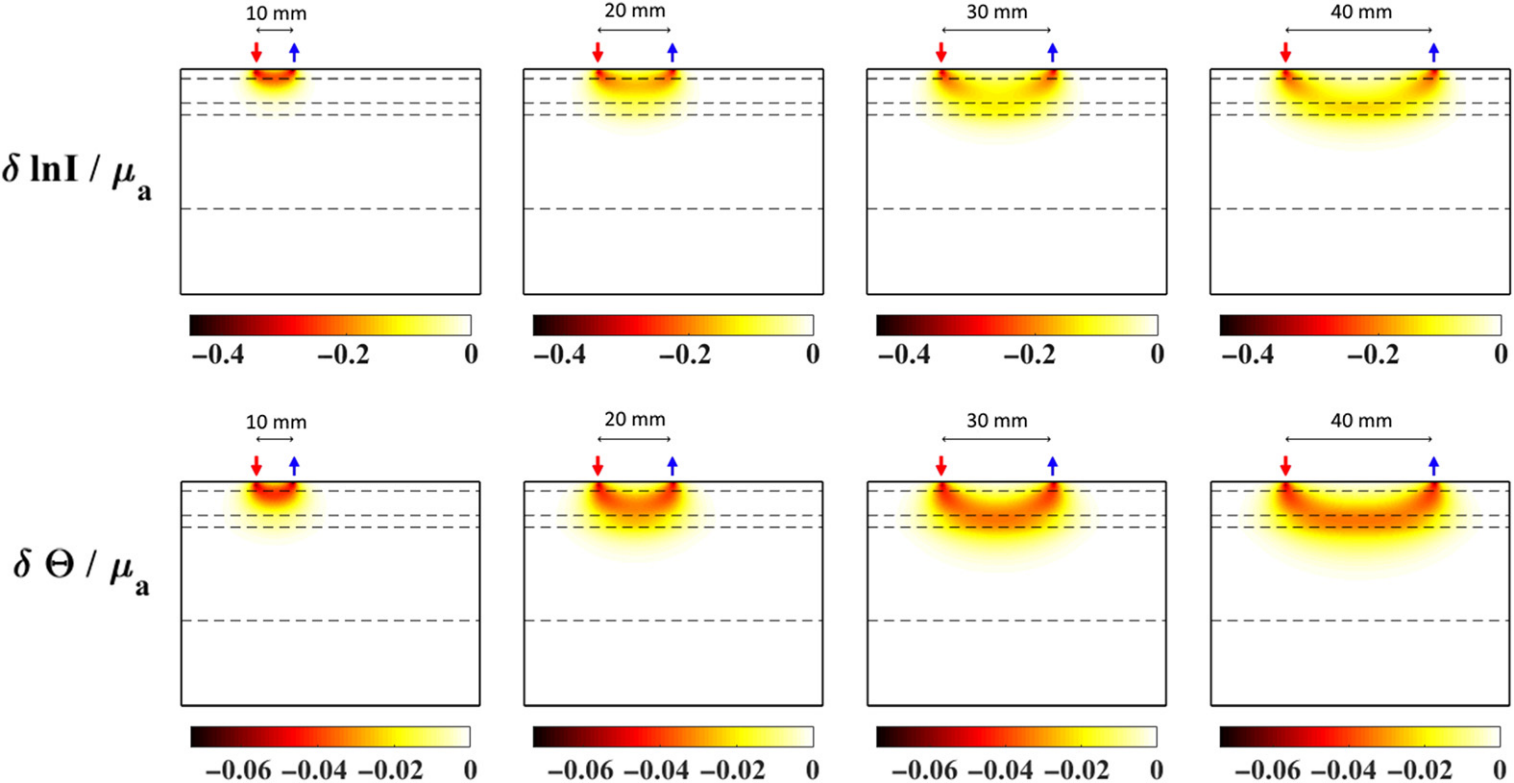
Jacobian of log intensity and phase measurements for an absorption coefficient at 830 nm, at SD distances of 10, 20, 30, and 40 mm, on a five-layered slab. The layers (dashed lines) from top to bottom represent skin/scalp, skull, CSF, gray matter, and white matter, with tissue properties shown in Table 1.

While CW-DOT is robust, inexpensive, and easy to use,^9^ the localization of focal activations is usually hindered due to the intensity-only measurements. However, at a relatively higher instrumentation cost as compared with CW systems, the use of a modulated NIR light typically at 100 to 200 MHz, known as a frequency domain (FD) system, enables the measurement of the phase shift of the detected NIR light after traversing through the tissue that corresponds to the average path length of the photon travelled. Recent work in FD-DOT^10^ has demonstrated the more uniform sensitivity of the phase measurements toward deeper tissue that results in a more accurate recovery of focal activations in the brain with a reduction of 59% in localization error and 21% effective resolution as compared with CW-DOT. To further demonstrate the benefit of utilizing phase measurements from an FD system, the sensitivities (Jacobian) of intensity (log intensity) and phase measurements at a single wavelength (830 nm) are shown in Fig. 1 for a five-layered slab model representing the skin, skull, CSF, gray matter, and white matter of the head. The thickness of the skin, skull, CSF, and gray matter is 2.5, 6.5, 3, and 25 mm, respectively, with the tissue properties as shown in Table 1, and the sensitivity maps are shown for SD distances of 10, 20, 30, and 40 mm. The increasing depth sensitivity of intensity and phase with increasing SD distance is clearly observed, and for the same SD distance, the phase measurement demonstrates a higher depth sensitivity, while being more uniform as compared with intensity.

**Table 1:** Background tissue properties (4).

Region	HbO (mM)	Hb (mM)	Sa (${\mathrm{mm}}^{-1}$)	Sp
Skin	0.0575	0.0313	0.53	1.15
Skull	0.0443	0.0195	0.72	0.89
CSF	0.0110	0.0083	0.30	0
Gray matter	0.0559	0.0350	0.50	1.73
White matter	0.0680	0.0265	0.81	1.31

While this is a very promising aspect of the FD-DOT technique, a straightforward superficial signal regression procedure on both intensity and phase signals, as will be shown, can result in misleading hemodynamic recovery. It is demonstrated in this work that, as the phase signals have relatively higher sensitivity toward deeper tissue than intensity signals, even the short distance phase measurements (which may unknowingly be used to regress superficial signal contamination) may be used to measure the functional activity from the deeper tissue.

The primary requirement of the regressor signal (the short distance measurement that is regressed from the measurements) is that it should be uncorrelated (i.e., orthogonal) to the signals originating at the deeper tissue. But in the case of phase, even the short distance measurement contains some deeper tissue information, making it correlated to the functional signal originating in the brain. Therefore, an unchecked regression of the phase signals using the short-distance phase measurement not only would include the unwanted superficial signal contamination in the recovery but also can result in a reduced component of the functional signal from the brain, which may lead to false positives of functional activations in the cerebral region. In this work, this phenomenon is demonstrated and an alternative regression methodology for reducing superficial signal contamination in the phase signal in the context of the FD DOT system is provided.

## 2 Superficial Signal Regression—Methodology

Consider an NIRS setup with M measurement channels of different SD combinations represented by the index i at time t to measure changes in intensity in log-scale $\Delta y_{i}(t)$, i.e., the change in attenuation (optical density) of intensity at a given wavelength. It can be expressed as a sum of changes in attenuation due to absorption changes in superficial tissue and in the brain tissue (assuming there are no scattering related changes), which is written as

$$\Delta y_{i}(t)=s_{ys}(i)\Delta \mu_{a_{s}}(t)+s_{yb}(i)\Delta \mu_{a_{b}}(t).$$ (1)

Here, $s_{ys}(i)$ and $s_{yb}(i)$ are the sensitivities of intensity with respect to absorption changes in superficial and brain tissues, respectively, corresponding to a SD channel-i. Similarly, the differential phase signal $\Delta p_{i}(t)$, has a contribution from absorption changes in superficial tissue and the brain tissue as

$$\Delta p_{i}(t)=s_{ps}(i)\Delta \mu_{a_{s}}(t)+s_{pb}(i)\Delta \mu_{a_{b}},$$ (2)

where $s_{ps}(i)$ and $s_{pb}(i)$ are the sensitivities of phase with respect to absorption changes in superficial and brain tissues, respectively, corresponding to channel-i. As seen in the above two equations, the NIRS signal is inherently contaminated with changes corresponding to the superficial tissue. In this regard, signal regression techniques are generally used to remove the hemodynamic changes from the superficial tissue, which can otherwise lead to artefacts or overshadow the cortical functional activation due to very high sensitivity of superficial tissue relative to the brain. The primary requirement to completely remove superficial signals using a regression method is that the changes in the superficial tissue and brain tissue must be orthogonal to each other.^11^ By definition, two signals $x_{1}(t)$ and $x_{2}(t)$ are said to be orthogonal if they are uncorrelated, i.e., their inner product $\langle x_{1},x_{2}\rangle =0$, which is defined as

$$\langle x_{1},x_{2}\rangle =\int_{-\infty }^{\infty }x_{1}(t)x_{2}(t)dt.$$ (3)

Considering $\Delta y_{nn}(t)$ to be the nearest-neighbor (short distance) intensity signal and assuming that it is strongly sensitive to the absorption changes in superficial tissue, i.e., $\Delta y_{nn}(t)=k\Delta \mu_{a_{s}}(t)$ (where k is the corresponding sensitivity factor), along with the condition that the absorption changes in superficial tissue are orthogonal to the absorption changes in brain tissue, i.e., $\langle \Delta \mu_{a_{s}},\Delta \mu_{a_{b}}\rangle =0$, the contribution from the superficial tissue is regressed using

$$\Delta y_{r_{i}}(t)=s_{yb}(i)\Delta \mu_{a_{b}}(t)=\Delta y_{i}(t)-\frac{\left\langle \Delta y_{i},\Delta y_{nn}\right\rangle }{\left\langle \Delta y_{nn},\Delta y_{nn}\right\rangle }\Delta y_{nn}.$$ (4)

For FD-NIRS systems, a similar regression method for phase-based phase regression is applied:

$$\Delta p_{r_{i}}(t)=s_{pb}(i)\Delta \mu_{a_{b}}(t)=\Delta p_{i}(t)-\frac{\left\langle \Delta p_{i},\Delta p_{nn}\right\rangle }{\left\langle \Delta p_{nn},\Delta p_{nn}\right\rangle }\Delta p_{nn}.$$ (5)

However, in the context of phase signals, this may not be directly applicable, as even the short distance phase signals will have some contribution coming from the deeper tissues, as shown in Fig. 1. This is due to the fact that the sensitivity of phase signals is higher for deeper tissues as compared with intensity. Therefore, implementing the regression as shown in Eq. (5), would lead to a decrease in the signal contrast from the brain and contaminate the brain signal with superficial tissue related changes. To avoid this, the use of short-distance intensity for the phase regression (intensity-based phase regression) is proposed:

$$\Delta p_{r_{i}}(t)=s_{pb}(i)\Delta \mu_{a_{b}}(t)=\Delta p_{i}(t)-\frac{\left\langle \Delta p_{i},\Delta y_{nn}\right\rangle }{\left\langle \Delta y_{nn},\Delta y_{nn}\right\rangle }\Delta y_{nn}.$$ (6)

The effect of these two signal regression methods will be demonstrated in the following sections through the simulation of visual cortical activation as observed using an FD-high density-DOT (FD-HD-DOT) measurement system. It will be clearly shown that a phase-based phase regression inaccurately retains the superficial signal even after regression, which will result in unwanted and potentially misinterpreted false positives of focal activations. The use of intensity-based phase regression is shown to be a much more accurate approach to fully removing the superficial signal contamination from the FD-HD-DOT reconstruction of focal activations using FD data.

## 3 Simulation

**Fig. 2:**
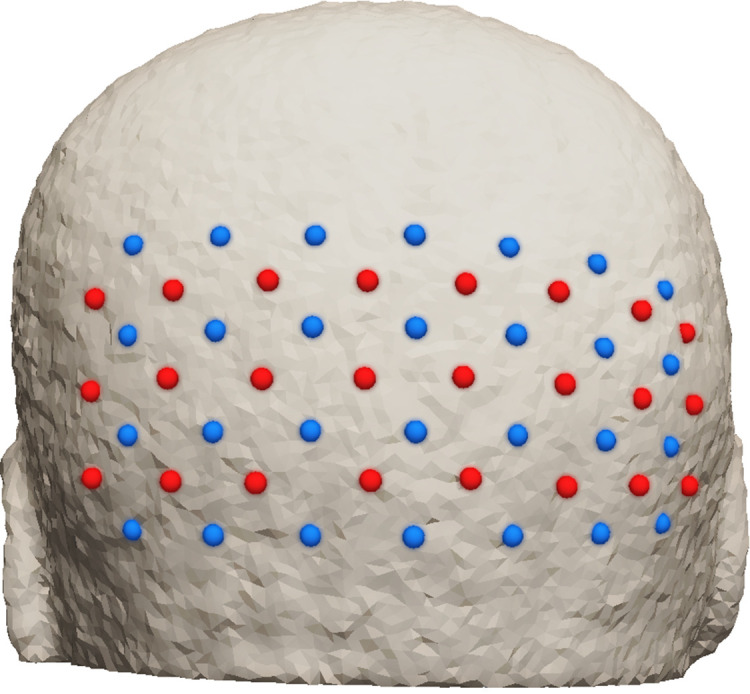
Array of 24 sources (red) and 28 detectors (blue) placed on the back of the head to probe the visual cortex region.

A realistic simulation of a functional activation in the visual cortex region is demonstrated in this section to show the effects of the abovementioned regression methods in the recovery of functional hemodynamic activity from the cerebral region. Consider a five-layer (skin, skull, CSF, gray matter, and white matter) head model mesh with 265 K nodes and $1.5\;{\mathrm{mm}}^{3}$ average volume of the tetrahedral elements and the tissue properties of oxy-hemoglobin (HbO) and deoxy-hemoglobin (Hb) concentration, scattering amplitude (Sa), and scattering power (Sp) as shown in Table 1 (4). The modeled measurement system consists of 24 sources and 28 detectors in a HD array, as shown in Fig. 2, which is placed on the back of the head to map visual-cortex activations. In such a grid pattern setup, SD distances can be categorized into different neighborhoods i.e., 13 (nearest neighbor 1 or NN1), 29 (NN2), 39 (NN3), 47 (NN4), 54 (NN5), and so on. Only the first four NNs are considered in this case as higher NNs are limited by the dynamic range of the detectors and the lower signal-to-noise ratio as previously reported.^10^

**Fig. 3:**
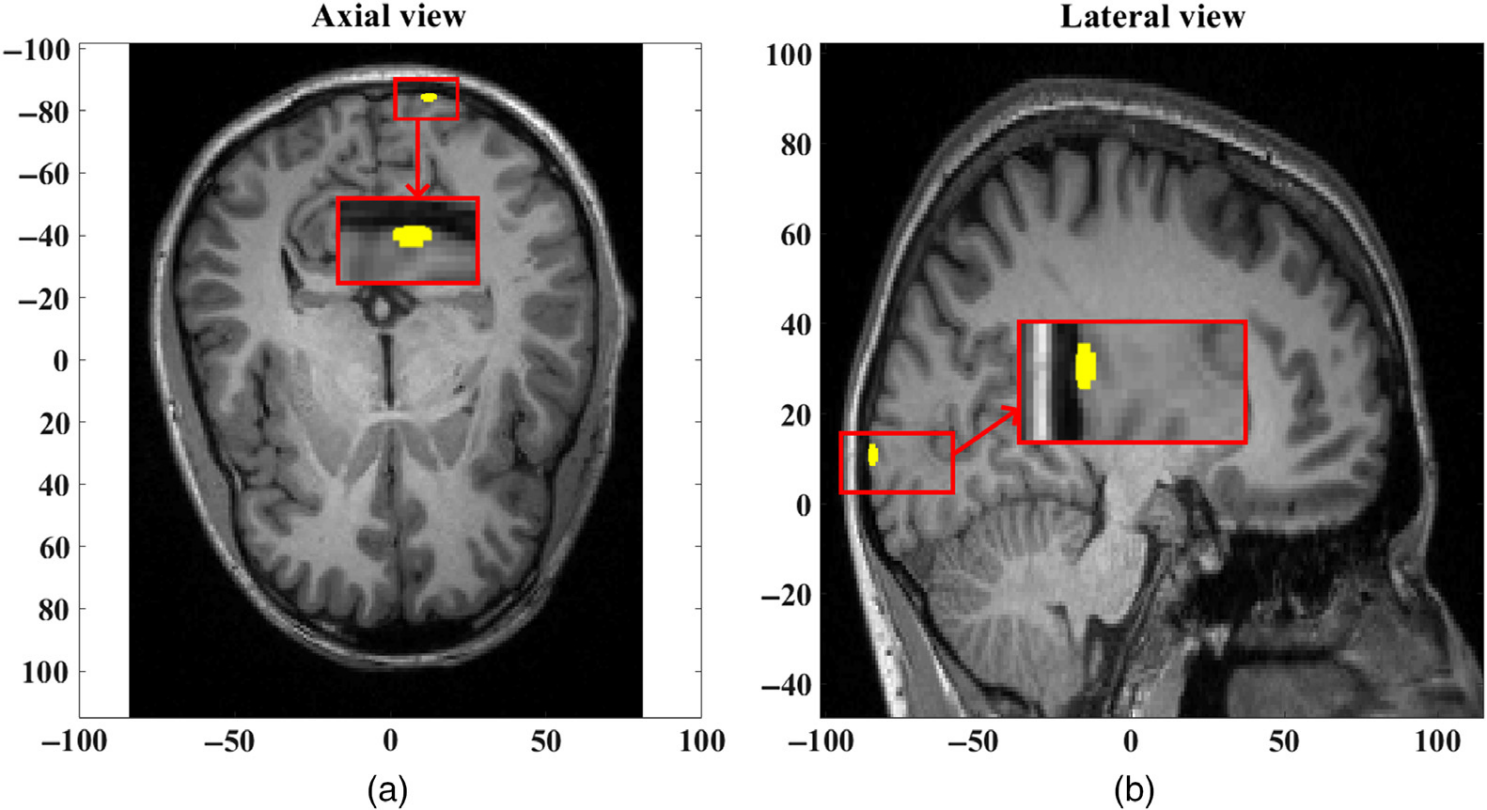
(a) Axial view and (b) lateral view of the focal activation in the gray matter at a depth of 10 mm and blob radius of 2.5 mm, at time t=10  s.

Physiological signals ($P_{j}(t)$) at 1.2, 0.25, and 0.1 Hz to model cardiac,^12^ respiratory,^13^ and Meyer waves,^14^ respectively, are added as sinusoidal changes^15^ in hemoglobin concentrations in skin, skull, gray matter, and white matter, along with a functional signal F(t) peaking at t=10  s originating within gray matter at a depth of 10 mm with an activation blob of radius 2.5 mm (Fig. 3). A superficial signal S(t) originating in the skin region, peaking at t=30  s, is also included to observe the effects of superficial layer signal contamination. These functional F(t) and superficial S(t) signals are modeled using a Gaussian distribution^16^ with their temporal full width half maximum (FWHM) values such that the two distributions are mutually separated in time to approximately represent two simple orthogonal signals for an ideal regression:

$$F(t)=F_{0}\;\exp(-{\left(\frac{t-10}{2}\right)}^{2}),$$ (7)

$$S(t)=S_{0}\;\exp(-{\left(\frac{t-30}{2}\right)}^{2}),$$ (8)

$$P_{j}(t)=P_{C_{j}}Q(\omega_{C},{\mathrm{BW}}_{C},t)+P_{R_{j}}Q(\omega_{R},{\mathrm{BW}}_{R},t)+P_{M_{j}}Q(\omega_{M},{\mathrm{BW}}_{M},t).$$ (9)

$$Q(\omega_{x},BW,t)=\underset{\omega }{\sum }A(\omega )\sin(\omega t+\xi ).$$ (10)

**Fig. 4:**
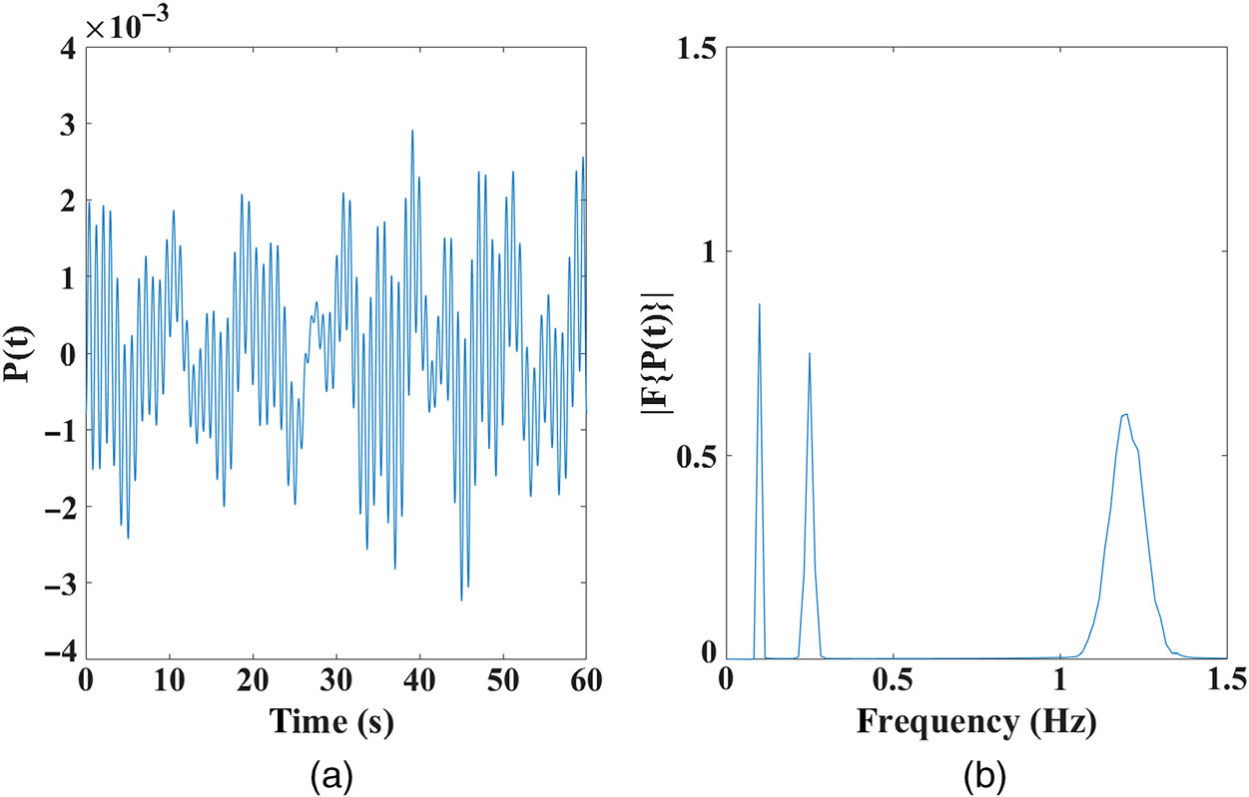
(a) Time trace and (b) frequency spectrum of physiological signal: a combination of cardiac, respiratory, and Meyer waves with 10% BW.

Here, $P_{j}(t)$ represents the combined physiological signals, the subscript j=1 represents skin and skull regions, and the subscript j=2 represents gray and white matter regions (assuming that CSF contains no physiological signal). The individual physiological signal $Q(\omega_{x},BW,t)$ is a sinusoidal signal with a non-zero bandwidth (“BW”) equal to $0.1\times \omega_{x}$, which is represented by a sum of sinusoidal signals given by Eq. (10), with their amplitude profile being centered at $\omega_{x}$, with a FWHM of the given BW, along with a random phase offset of ξ. This ensures that the function Q lies between −1 and 1 with $P_{C_{j}}$, $P_{R_{j}}$, and $P_{M_{j}}$ representing the respective amplitudes of cardiac, respiratory, and Meyer waves from Eq. (9), respectively. The physiological signal P(t), along with its frequency spectrum showing a 10% BW associated with each signal, is shown in Fig. 4.

All of the signals from Eqs. (7) to (9) represent a percentage change to the background HbO concentration as given in Table 1 with the corresponding Hb change considered to be of the same magnitude but opposite sign. For the focal activation, however, it is known that the HbO and the total hemoglobin concentrations increase while the Hb decreases.^17^ In line with this, the Hb change for focal activation is considered to be −0.47 times the change observed in HbO.^10^ The amplitude $F_{0}$ is chosen such that the corresponding maximum observable change in intensity is 0.05 in log scale to accurately represent experimental measurements.^18^ While the functional signal F(t) is only confined to the activation blob of 2.5 mm, the superficial signal S(t) occurs in the entire skin region and therefore has a quantitatively higher effect on the measured signals. In this regard, $S_{0}$ is chosen to be 100 times lower than $F_{0}$ so that both functional and superficial signals can be clearly observed in both intensity and phase. Effectively, the values of the signal strengths considered in this simulation are $P_{C_{1}}=0.0018$, $P_{C_{2}}=0.006$, $P_{R_{1}}=9\times 10^{-4}$, $P_{R_{2}}=0.0015$, $P_{M_{1}}=7\times 10^{-4}$, $P_{M_{2}}=0.0012$, $F_{0}=3$, and $S_{0}=0.03$.

**Fig. 5:**
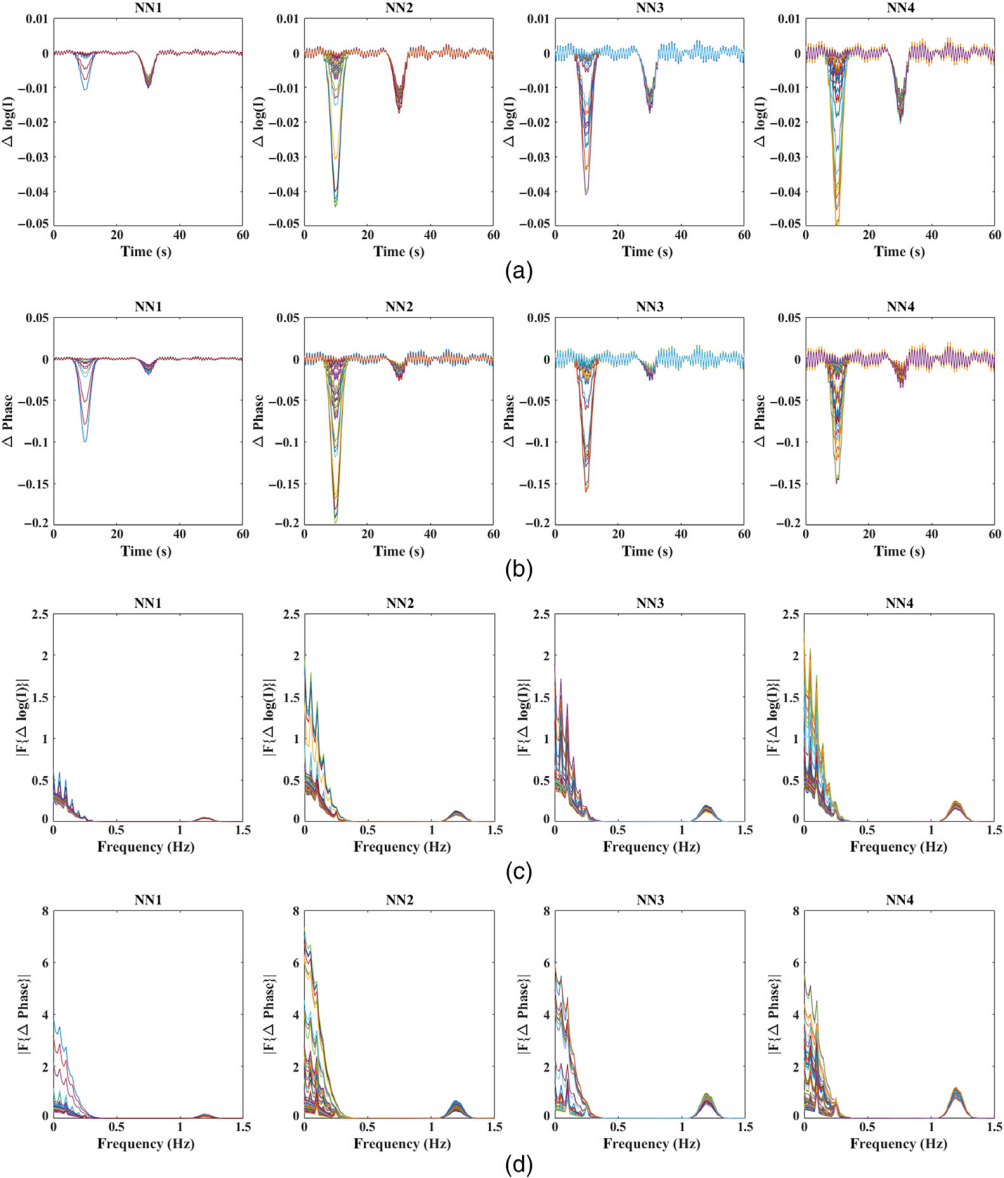
(a) and (b) Time traces of intensity and phase measurements without noise at four NNs modeled at 830 nm and (c) and (d) respective frequency spectra of intensity and phase measurements.

The data are simulated at a 40-Hz sampling rate, at wavelengths 830 and 690 nm and an intensity modulation frequency of 140 MHz using NIRFAST,^19^ with the resulting intensity and phase measurements for all source/detector measurements along with their frequency spectrum shown in Fig. 5. The low-frequency Gaussian profile with ripples at the 0.05 Hz (= 1/20) interval simply indicates that the two Gaussian peaks in the signal at an interval of 20 s correspond to functional and superficial signals in this study and the peaks at 0.1, 0.25, and 1.2 Hz correspond to the physiological signals. In accordance with Eqs. (7) and (8), the negative peak at t=10  s in the data as shown in Figs. 5(a) and 5(b) corresponds to the increase in HbO and decrease in Hb concentrations at t=10  s. This is the functional signal from the modeled focal activation, and it becomes stronger (particularly for intensity data) with increasing SD distance owing to the higher depth sensitivity at higher NN distance. The data even at higher SD separations are also seen to be contaminated with superficial signal as identified by the peak at t=30  s. The varying amplitudes of functional and superficial signals at different SD distances in Figs. 5(a) and 5(b) are due to the varying sensitivity of intensity and phase measurements at different SD distances.

**Fig. 6:**
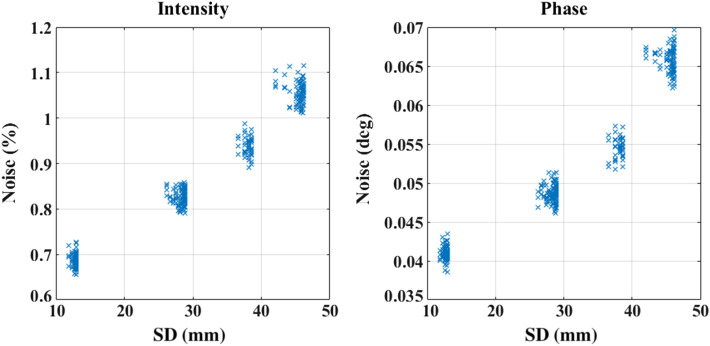
Noise levels of intensity and phase measurements as a function of SD distances.

Gaussian random noise is added to the simulated measurements as a function of SD distances to model realistic data based on an empirically derived noise model. The empirical noise model was built on data measured using ISS Imagent™ (an FD system console), with six detectors at distances of 18 to 58 mm from the source, placed on the visual cortex of a healthy subject. The source modulation frequency was set at 140 MHz, and data was recorded at a sampling rate of 39.74 Hz while the subject was at rest and quietly fixated on a blank screen. The noise was then estimated as the standard deviation of log mean intensity measurement as intensity noise and of phase difference (in deg) as the phase noise. It was then fitted using a two-term exponential as a function of SD distance details, which can be found elsewhere.^10^ The derived noise levels are plotted in Fig. 6 for the four NN SD distances in the measurement setup as shown in Fig. 2.

**Fig. 7:**
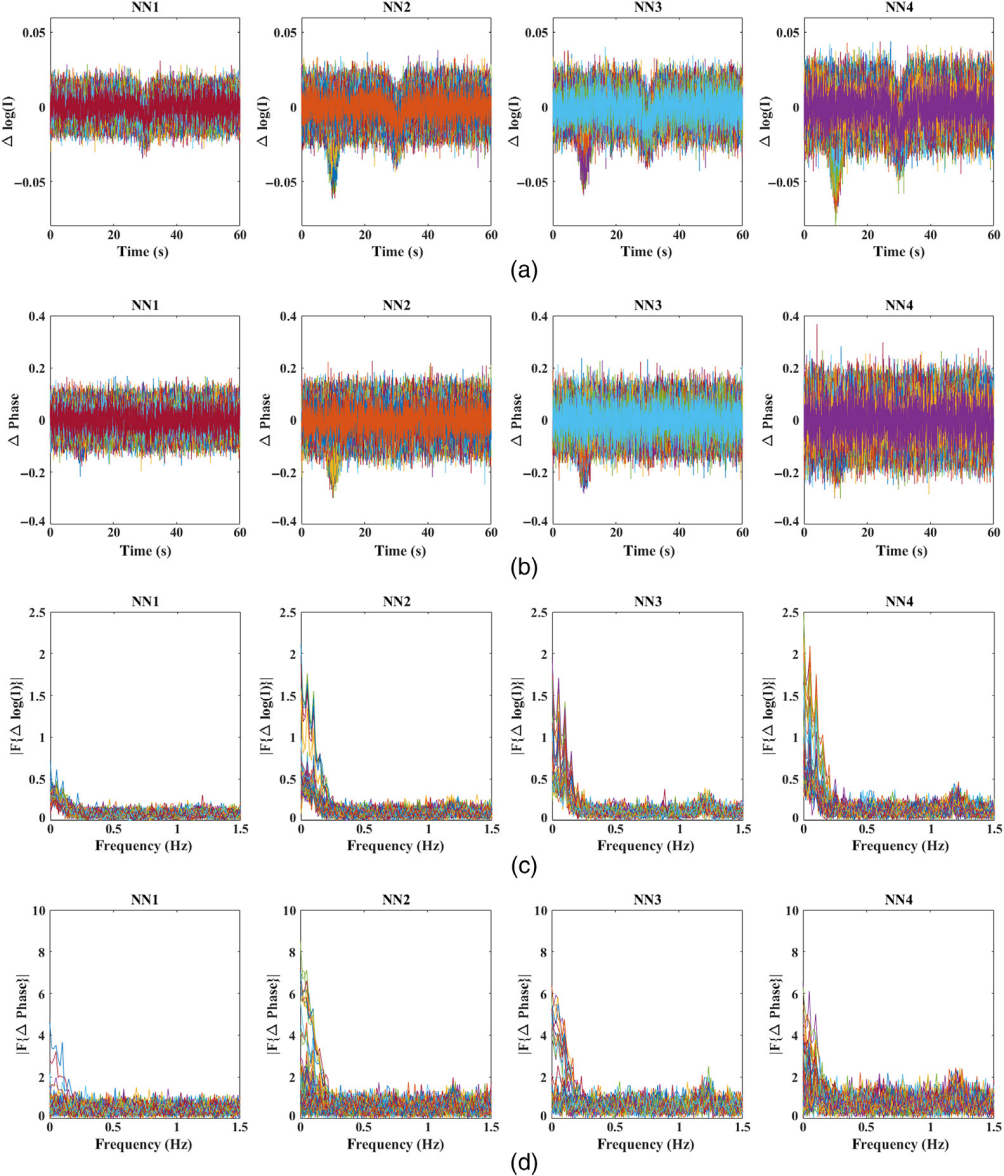
(a) and (b) Time traces of intensity and phase measurements with noise added at four NNs modeled at 830 nm and (c) and (d) respective frequency spectra of intensity and phase measurements.

The raw intensity and phase signals with the added noise at 830 nm for multiple SD distances (grouped as neighborhoods) are shown in Fig. 7.

To reduce the noise in the modeled data, similar to a realistic experimental procedure, the following pre-processing steps are applied before the superficial signal regression procedure:

a. The measurements are high-pass filtered at a 0.01 Hz cutoff frequency to remove any drifts present in the signal caused due to the measurement systems (not directly applicable in this simulation experiment).^10^
b. Measurements are then low-pass filtered at a 0.1 Hz cutoff frequency to remove the physiological noise, i.e., pulse, respiratory, and Meyer waves.^10^
c. Data are then down-sampled from 40 to 1 Hz with averaging every 40 samples, thereby greatly reducing random noise present in the signals.
d. To improve the contrast and further reduce noise, the measurements from multiple repetitions of similar excitations are block-averaged.^20^ In this case, 10 repetitions are averaged together to increase the signal-to-noise ratio of the data.

While the regression of intensity data is well understood,^18^ the regression of phase signals is the primary objective of this work. It is observed from Figs. 5 and 7 that the functional signal is also seen in the first NN (NN1) measurements of phase, while the NN1 measurements of intensity signals contain predominantly the superficial signal. Therefore, the following two regression methods are implemented on the phase data:

a. regression of phase signals with the short-distance phase signal (phase-based phase regression).
b. regression of phase signals with the short distance intensity signal (intensity-based phase regression).

**Fig. 8:**
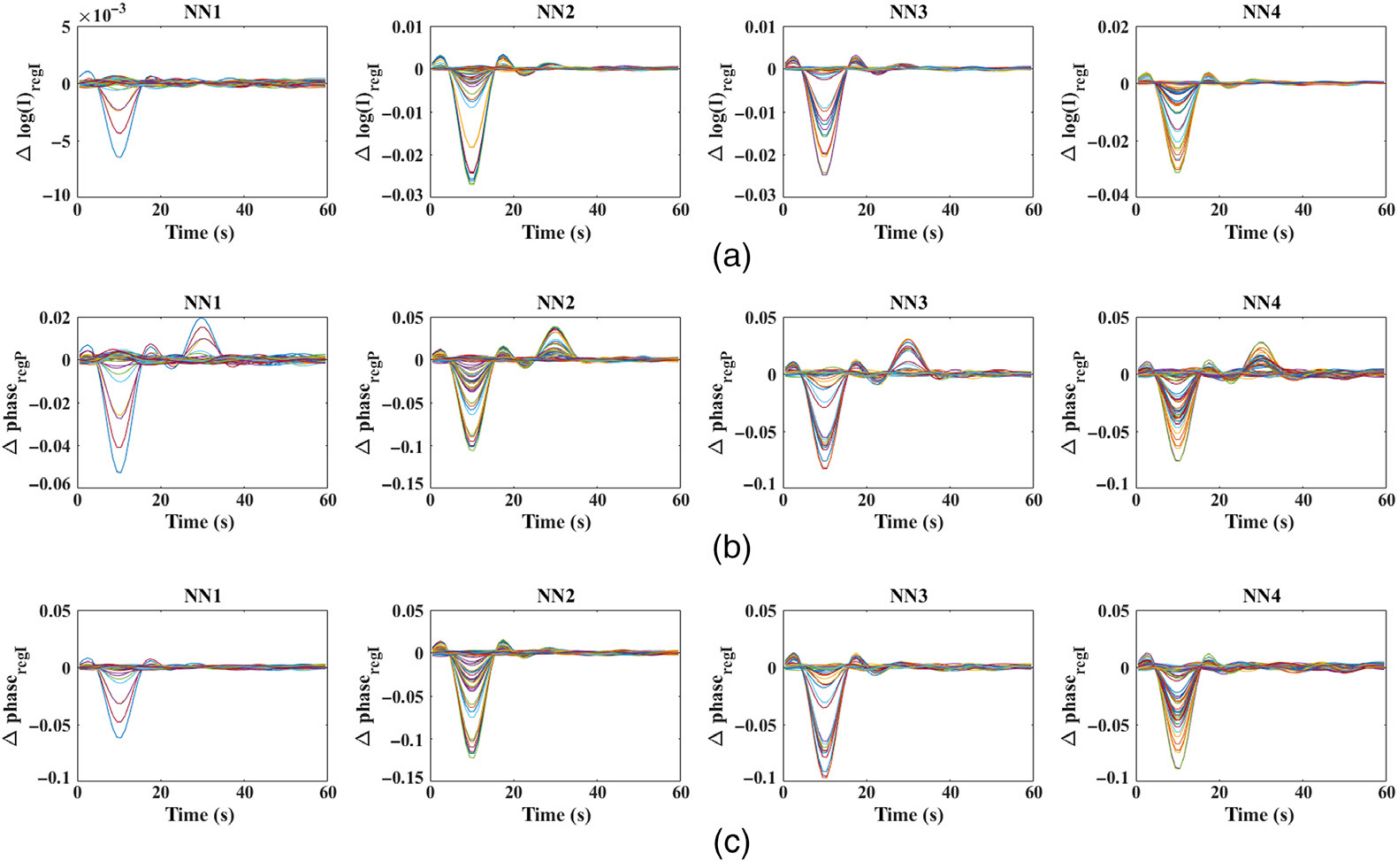
(a) Regressed intensity measurements with short-distance intensity measurement; (b) regressed phase measurements with short-distance phase measurement; and (c) regressed phase measurements with short-distance intensity measurement post filtering and all noise-reduction steps.

The regressor signal (short-distance measurement) is regarded as the average of all NN1 measurements of either intensity or phase corresponding to the regression method. The result of regression methods on this processed and noise-reduced data is shown in Fig. 8.

It can be seen from Fig. 8(a), that the contamination due to the superficial signal at t=30  s is clearly removed from intensity data after regression with short-distance intensity data. The phase signals, however, retained the superficial signal contamination (t=30  s) with a flipped sign after the regression with the short-distance phase measurement, as seen in Fig. 8(b). The regression procedure finds the overlap (cross-correlation coefficient) between each phase measurements and the average of NN phase measurement ($\Delta p_{nn}$) and thus subtracts $\Delta p_{nn}$ of the amplitude proportional to this overlap as defined in Eq. (5). As $\Delta p_{nn}$ has both a superficial signal and a functional component, any phase measurement with a higher relative strength of functional to superficial signal, as compared with $\Delta p_{nn}$, would result in an overlap value greater than the individual strength of superficial signal present. Therefore, subtracting a higher amplitude of superficial signal than what is present, we observe a change in sign for superficial signals after regression with $\Delta p_{nn}$. However, with the regression of phase signals using short-distance intensity measurement, the superficial signal contamination is clearly seen to be removed in Fig. 8(c).

To quantitatively represent the reduction of superficial signal contamination, before and after regression, a correlation coefficient between the measurement $\Delta g_{i}(t)$ and the superficial signal S(t) are calculated as follows:

$$R(g_{i},S)=|\frac{\left\langle \Delta g_{i},S\right\rangle }{\sqrt{\left\langle \Delta g_{i},\Delta g_{i}\right\rangle }\sqrt{\left\langle S,S\right\rangle }}|,$$ (11)

where g can represent either intensity $\Delta y_{i}(t)$, or phase $\Delta p_{i}(t)$, and the index i represents a measurement channel corresponding to a SD combination. The value of $R(g_{i},S)$ can vary from 0 to 1 (equivalent to 0% to 100%), which directly represents the amount of superficial signal S(t) present in each measurement $\Delta g_{i}(t)$.

**Fig. 9:**
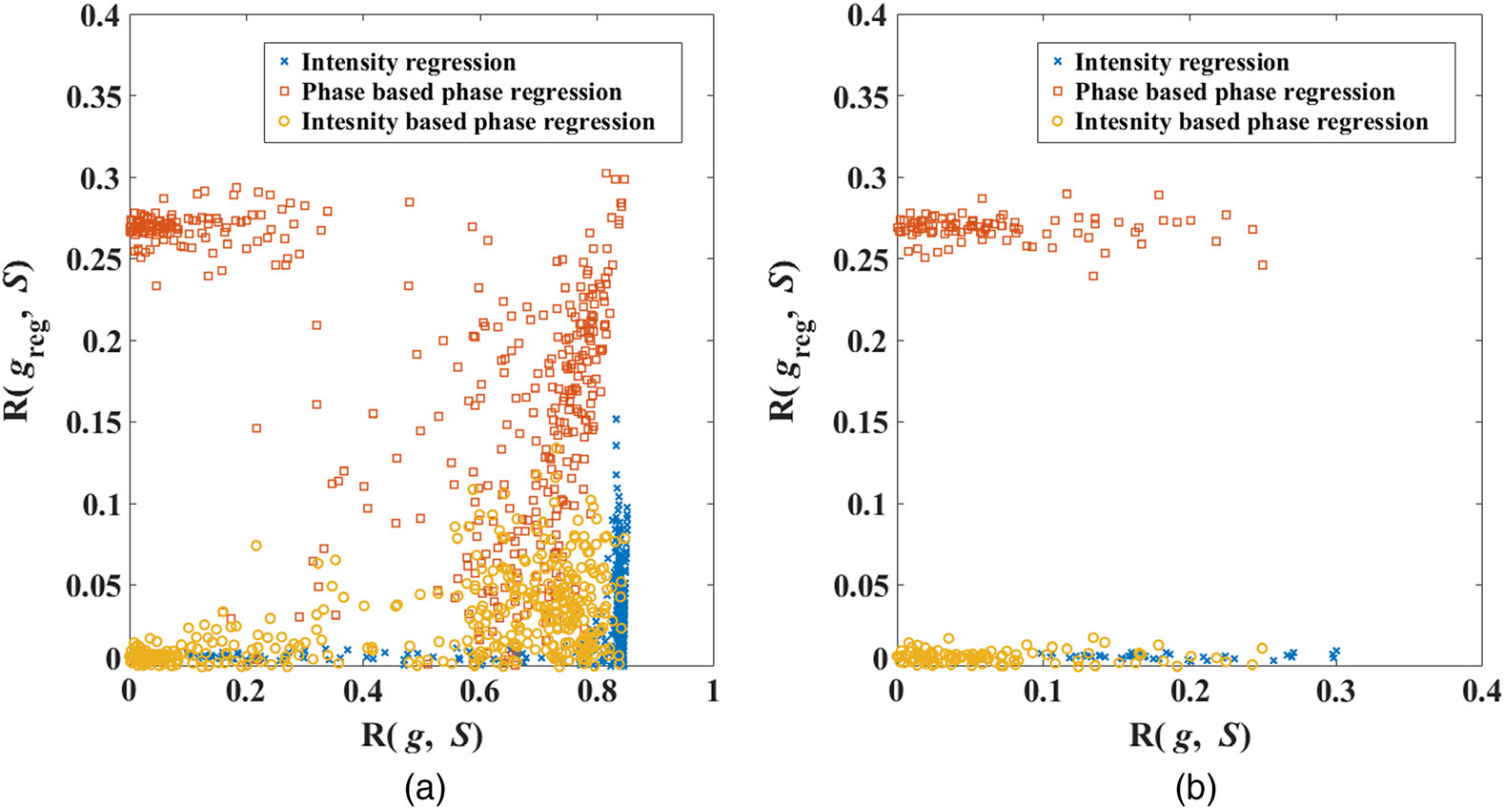
Correlation coefficient of all intensity and phase measurements with respect to superficial signal, before superficial signal regression vs after superficial signal regression, for (a) all measurement channels (b) for the channels detecting 90% and above of the maximum functional activity.

To further observe the amount of superficial signal contamination present exclusively in a subset of the channels that substantially detect the functional activity, a threshold of 90% is considered over the maximum value of $R(g_{i},F)$, i.e., $R(g_{i},F)>0.9\times \max\{R(g_{i},F)\}$, where F(t) is the functional signal and the value $R(g_{i},F)$ defines the amount of functional signal present in the measurement, similar to Eq. (11). The correlation coefficients $R(g_{i},S)$ for all of the measurements before and after regression are shown in Fig. 9(a), and the correlation coefficients for the channels that detect functional activity are shown in Fig. 9(b).

As the value of the correlation coefficient for all of the measurement channels varies from 0 to 1, a root mean square (RMS) value of $R(g_{i},S)$ over all measurement channels i (or a subset of channels that detect functional activity) is considered to represent an effective strength of the superficial signal in the measurements of either intensity or phase and is shown in Table 2 before and after the regression procedure.

**Table 2:** RMS values of correlation coefficients indicating the amount of superficial signal contamination in the intensity and phase measurements before and after regression at 830 nm (the values within brackets correspond to 690 nm).

	Measurement type	Before regression $R(g_{i},S)$	After regression $R(g_{{\mathrm{reg}}_{i}},S)$	Suppression factor
All measurement channels	Intensity	0.7774 (0.8174)	0.0361 (0.0479)	21.5 (17.1)
	Phase	0.5881 (0.5572)	0.2072 (0.0567) (phase-based regression)	2.8 (9.8)
			0.0402 (0.0420) (intensity-based regression)	14.6 (13.2)
Channels detecting at least 90% of the functional activity	Intensity	0.1944 (0.4882)	0.0102 (0.0375)	18.9 (13.0)
	Phase	0.0797 (0.1168)	0.2807 (0.0544) (phase-based regression)	0.3 (2.1)
			0.0107 (0.0360) (intensity-based regression)	7.4 (3.2)

The suppression factors in Table 2 correspond to the ratio of correlation coefficient $R(g_{i},S)$ before and after regression, and a value >1 implies that the regression procedure has reduced the superficial signal contamination. The suppression factor for intensity-based phase regression is not only >1 but also higher than the phase-based phase regression procedure, therefore indicating a better reduction in the superficial signal contamination. A value <1 of the suppression factor for phase-based phase regression for the channels that detect functional activity highlight that this regression procedure increased the amount of superficial signal contamination in the channels that detect functional activity. The effect of this false-positive suppression will be better demonstrated through tomographic reconstruction.

Tomographic reconstruction of hemoglobin concentration changes based on the regressed intensity and phase data as shown in Fig. 8 is performed. The Jacobian J (the sensitivity of intensity and phase with respect to the absorption coefficient at every node in the head model) is constructed^21^ with background optical properties as given in Table 1 on a measurement setup as shown in Fig. 2 at two wavelengths of 690 and 830 nm, individually, to retrieve the respective absorption changes at these wavelengths. The retrieved absorption change at each wavelength is then related to the HbO and Hb concentration changes:^22^

$$[\begin{array}{c} {\Delta \mu }_{a690}\\ \Delta \mu_{a830} \end{array}]=[\begin{array}{cc} \varepsilon_{\mathrm{HbO},690} & \varepsilon_{\mathrm{HbO},830}\\ \varepsilon_{\mathrm{Hb},690} & \varepsilon_{\mathrm{Hb},830} \end{array}][\begin{array}{c} \Delta \mathrm{HbO}\\ \Delta \mathrm{Hb} \end{array}].$$ (12)

The absorption changes ($\Delta \mu_{a}$) at every node of the head model is related to the measurement changes (Δy and Δp), and the sensitivity matrix is as follows:

$$[\begin{array}{c} \Delta y\\ \Delta p \end{array}]=J.\Delta \mu_{a}=[\begin{array}{c} J_{y}\\ J_{p} \end{array}]\Delta \mu_{a}.$$ (13)

The Jacobian J consists of both the sensitivity of intensity ($J_{y}$) and phase sensitivities ($J_{p}$), which are different in their magnitude.^10^ To retrieve the absorption changes, a single step inversion of the Jacobian is performed together with Tikhonov regularization to compensate for the problem being ill-posed and ill-conditioned.^19^

$$\Delta \mu_{a}=J^{∼1}[\begin{array}{c} \Delta y\\ \Delta p \end{array}]=J^{T}{\left({JJ}^{T}+\alpha I\right)}^{-1}[\begin{array}{c} \Delta y\\ \Delta p \end{array}].$$ (14)

Here, α is the regularization factor, which is considered to be $\alpha =\Lambda \;\max[\mathrm{diag}({jj}^{T})]$, with the weight factor Λ=0.01 to smooth the parameter recovery.^20^ The regularization is implemented separately for the intensity Jacobian and phase Jacobian kernels.^10^ The recovered absorption changes at two wavelengths are then used to retrieve the corresponding changes in hemoglobin concentrations using Eq. (11).

**Fig. 10:**
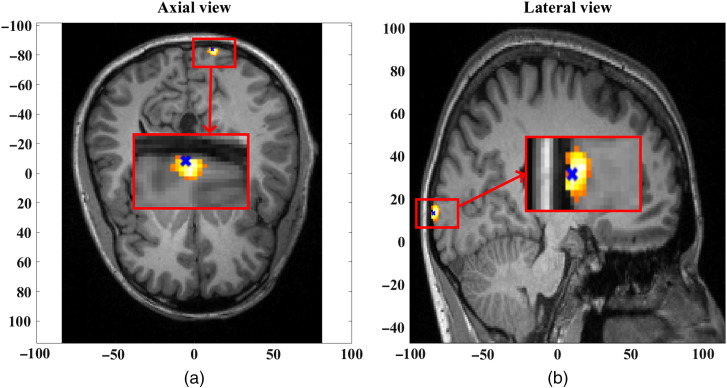
Axial view at (a) z=11.8  mm and (b) lateral view at x=12.5  mm of the recovered HbO at t=10  s showing a positive change, reconstructed using the intensity data regressed with short-separation intensity measurement and phase data regressed with short-separation phase measurement. The center of ground-truth focal activation at (11.5, −85, and 10.7) is shown by the blue “x” mark.

The recovered focal activation using the intensity data regressed with short-separation intensity measurement (intensity-based intensity regression) and phase data regressed with short-separation phase measurement (phase-based phase regression) at t=10  s is shown in Fig. 10. This is achieved by spatially thresholding the recovered hemoglobin concentration at 50% of the maximum change. The size of the observed activation in Fig. 10 indicates the FWHM of the recovery, which is found to be 10.1 mm given the 5 mm (2.5-mm radius) of ground-truth focal activation. Similar recovered focal activation is observed using the intensity data and phase data regressed with short-separation intensity measurement (intensity-based intensity regression and intensity-based phase regression), at t=10  s, also with an FWHM of 10.1 mm.

**Fig. 11:**
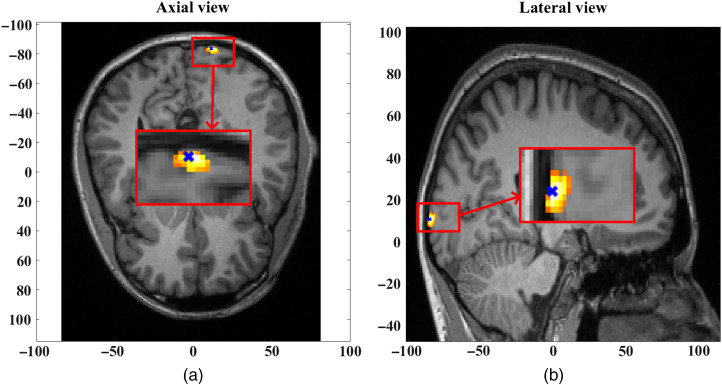
(a) Axial view at z=9.8  mm and (b) lateral view at x=13.5  mm of the false-positive recovery of HbO at t=30  s showing a negative change, reconstructed using the intensity data regressed with short-separation intensity measurement and phase data regressed with short-separation phase measurement. The center of ground-truth focal activation (11.5, −85, and 10.7), occurring at t=10  s is shown by the blue x mark.

However, at t=30  s, the recovered hemoglobin changes show a “false” focal activation for the phase-based phase regression method, as shown in Fig. 11, indicating a false positive. The maximum absolute change observed for the recovered HbO concentration is seen as a negative change, as shown in the data in Fig. 8. The focal activation recovered and shown in Fig. 11 indicates a negative change of HbO concentration thresholded at 50% of the maximum absolute change, with an FWHM of 9 mm. The HbO recovery for intensity-based phase regression at t=30  s, however, does not show any specific focal activation recovery, which is also in line with the data observed in Fig. 8.

**Fig. 12:**
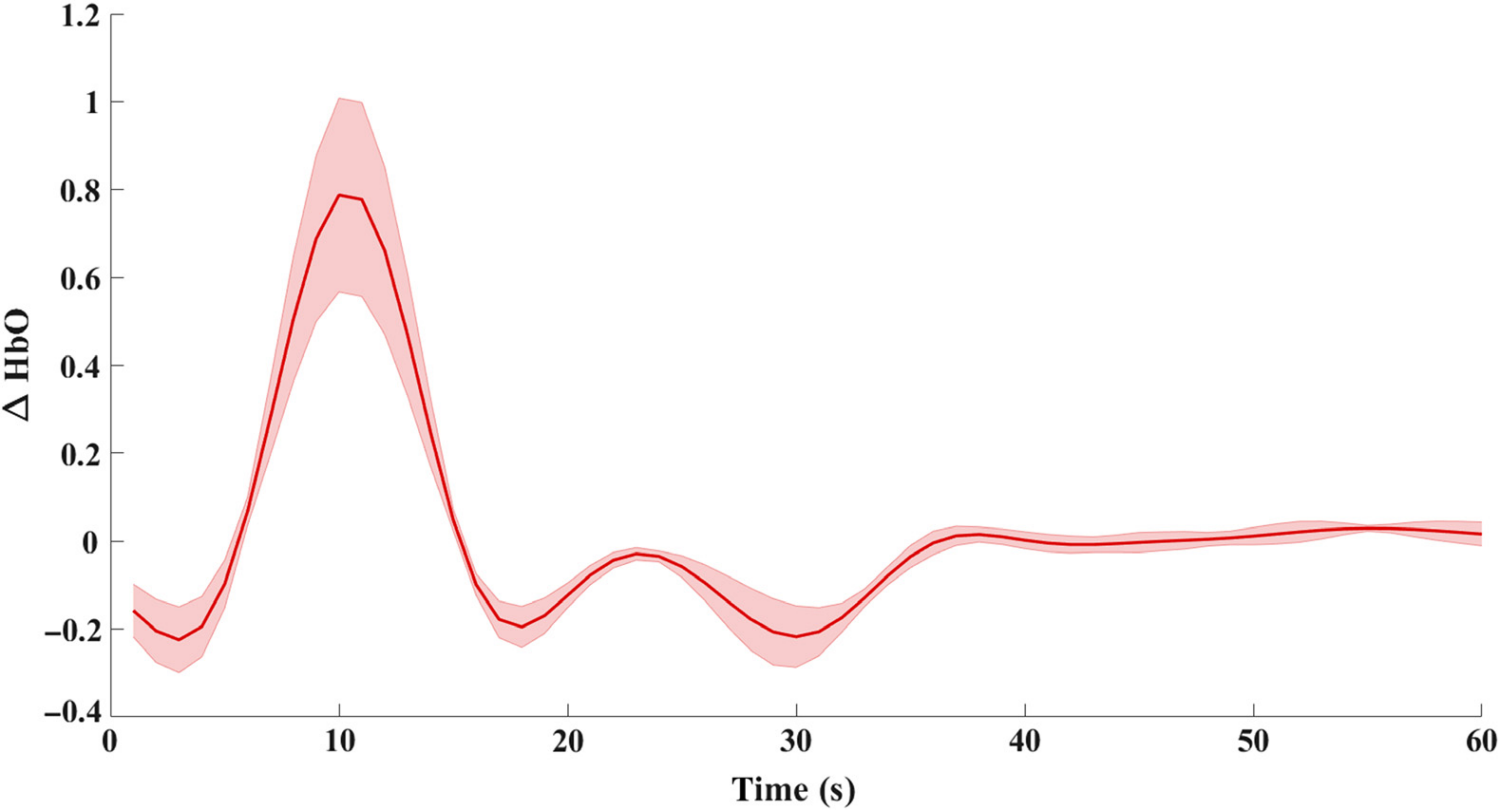
Temporal plot of normalized HbO recovery in the region of ground-truth focal activation for the phase-based phase regression method.

**Fig. 13:**
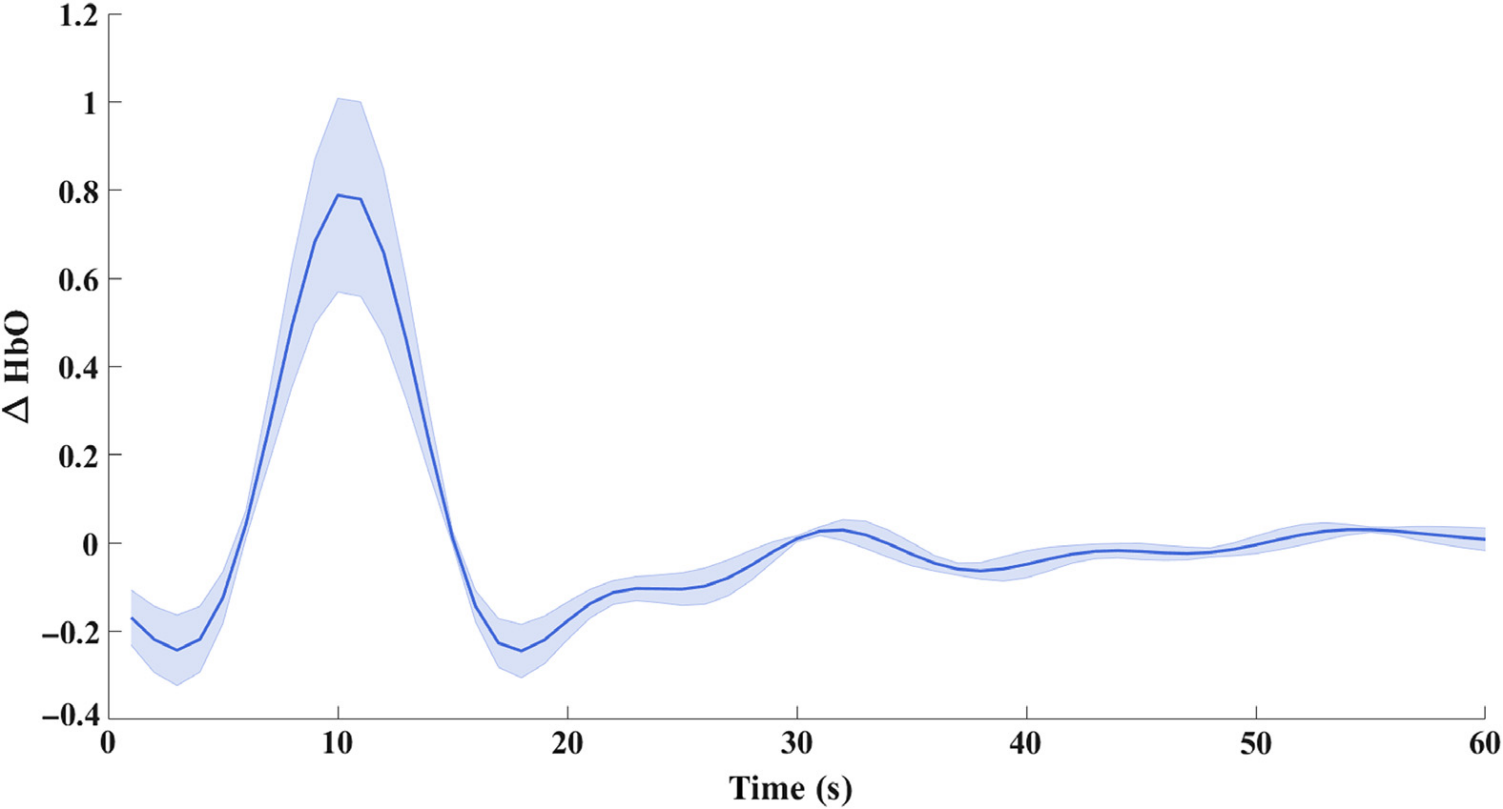
Temporal plot of normalized HbO recovery in the region of ground-truth focal activation for the intensity-based phase regression method.

To further substantiate the presence of superficial signal contamination in phase-based phase regression in comparison with the intensity-based phase regression, the recovered HbO in the region of ground-truth activation, i.e., within a 2.5-mm radius from (11.5, −85, and 10.7) is shown in Figs. 12 and 13. The negative side lobes on either side of the peak at t=10  s is simply an effect of using the band-pass filter on a Gaussian signal. The negative peak of HbO in Fig. 12, at t=30  s, clearly indicates the direct effect of superficial signal contamination introduced due to the phase-based regression method appearing not only in the cerebral region but also in the locality of ground-truth activation, therefore leading to false positives.

## 4 Discussion

Recent developments in FD-DOT imaging studies have shown that the use of phase along with intensity data significantly improves the image quality. The higher depth sensitivity of phase signal relative to intensity signal enables a significantly better localization of focal activations especially in depth using an FD-DOT system, out-performing a CW-DOT system. However, performing similar pre-processing techniques of signal regression that are implemented in a CW-DOT system without careful observation will lead to unintended and misleading hemodynamic activity and therefore misinterpretation of the results.

While the superficial signal regression for intensity data is well understood and utilized often to improve the image quality, the signal regression for phase data is not as straightforward or well understood. The very advantageous aspect of higher sensitivity of phase signal to deeper tissue can lead to even the short-distance phase measurement detecting functional activity from deeper tissue. Therefore, any regression of phase signal using this short-distance phase measurement (phase-based phase regression) would not completely remove the superficial signal contamination and may additionally cause a reduction in the strength of the recovered functional signal. To this end, superficial signal regression of phase data using short-distance intensity measurement (intensity-based phase regression) is shown to more accurately remove the superficial signal contamination.

Utilizing realistic head model data, Fig. 5(b) highlights the presence of the functional signal, originating in the cerebral region at a depth of 10 mm from surface, even in the short-distance phase signals, while the short-distance intensity signals mostly contain the superficial signal. Therefore, given the superficial signal being uncorrelated to the functional signal, the regressor signal in the phase-based phase regression method (i.e., short-distance phase measurement) is still correlated with the functional signal. This is unlike the regressor signal in the intensity-based phase regression method (i.e., short-distance intensity measurement), which is uncorrelated to the functional signal and affects the regression procedure as highlighted in Sec. 2. This is seen in the results of Fig. 8(b), where an unbalanced regression using short-distance phase measurement causes a negative effect of signal contamination (inverted peak at t=30  s) in the phase data. It is also seen to reduce the functional component in the signals [decreased magnitude at t=10  s, as compared with raw data in Fig. 5(b)] that can cause a reduction in the contrast of recovered functional activation; the stronger the functional component in the regressor signal (short distance phase measurement) is, the greater the reduction in the functional component is in all of the measurements post regression. But using short distance intensity measurement to regress phase in Fig. 8(c) has proven to be efficient in its performance by both retaining a higher functional component and suppressing the superficial signal component at t=30  s.

The strength of the superficial signal present in intensity and phase measurements as defined by the correlation coefficient shown in Fig. 9 indicates that the phase-based phase regression retains a higher strength of the superficial signal in the regressed data as compared with intensity regression and intensity-based phase regression procedures. The RMS value of the correlation coefficient (calculated on all measurement channels) shown in Table 2, before and after the regression, indicates the reduction in superficial signal contamination at 830 nm by a factor of 2.8 for phase-based phase regression (13% of what is observed in intensity regression) and 14.6 for intensity-based phase regression (68% of what is observed in intensity regression), demonstrating the improvement in the regression of phase measurements using short-separation intensity measurement. The corresponding suppression factors at 690 nm are 9.8 for phase-based phase regression (57% of what is observed in intensity regression) and 13.2 for intensity-based phase regression (77% of what is observed in intensity regression). The relatively higher suppression factors for phase-based phase regression at 690 nm compared with those at 830 nm is because the functional signal present in the short-separation phase measurement at 690 nm is relatively lower than that at 830 nm, therefore making the phase regressor signal more uncorrelated to the functional signal. The lower functional activity at 690 nm corresponds to the lower Hb change i.e., 0.47 times that of HbO change, as the signal at 690 nm is more sensitive to Hb and at 830 nm it is more sensitive to HbO.

The DOT parameter recovery based on this regressed data is seen in Fig. 10. The focal activations at t=10  s for both phase-based phase regression and intensity-based phase regression methods (together with regressed intensity data in both cases) are found to have maxima location at a distance of 2.2 mm from the center of ground-truth activation, with a FWHM of 10.1 mm corresponding to a ground-truth width of 5 mm of the focal activation. At t=30  s, however, the DOT parameter recovery of hemodynamic activity for the phase-based phase regression method, as seen in Fig. 11, estimated a false-positive focal activation at maxima location also at a distance of 2.2 mm from the center of ground-truth focal activation (that occurred only at t=10  s) and an FWHM of 9 mm.

The cause of such a false positive can be explained by considering a set “S” of measurement channels that prominently detect the functional signals that originate in the region R. Conversely, any change in these S set of measurement channels corresponds to a prospective recovery in the region R. The regressor signal of phase, which contains both functional and superficial signal content, would cause a new peak corresponding to the superficial signal, if it is absent initially in the given S set of measurements, which would therefore result in a recovery in the original activation region “R.” Thus, instead of removing superficial signal contamination, the phase-based regression approach would cause the superficial signal to directly contaminate the hemodynamic recovery in the cerebral region. This is further substantiated by Figs. 12 and 13, which show the recovered HbO concentration over the entire period of time in the original focal-activation region R, where it is seen that a negative peak at t=30  s for the phase-based phase regression method shows the superficial signal contamination, while the HbO recovery for the intensity-based phase regression method did not show any such peaks at t=30  s.

## 5 Conclusion and Future Work

This work has demonstrated the adverse effects of implementing a phase-based phase regression in FD-DOT and shows the existence of superficial contamination in such cases even after regression, due to the possibility of the presence of the functional signal in phase data even at shorter SD separations, while also reducing the functional component in all of the measurements, which can lead to a reduction in the contrast of recovered focal activations. An alternative to the intensity-based phase regression method is proposed and demonstrated to correctly remove superficial signal contamination. This type of regression plays a significant role in any FD-based methods in which signal regression is implemented, such as FD-DOT. The same theory can also be extended for time resolved systems (TRS) in which the moments-based analysis is implemented to observe hemodynamic activity, as the first-order moment of the distribution of time of flight of photons is similar to the phase measurement in FD NIRS systems and can also become significant to other data types (higher order moments in TRS) that have a higher sensitivity toward deeper tissue and therefore require an intensity-based (total photon count in TRS) regression approach instead of using the same data type for superficial signal regression. This needs further investigation and is the subject of future studies.
